# Effects of Native Banana Starch Supplementation on Body Weight and Insulin Sensitivity in Obese Type 2 Diabetics

**DOI:** 10.3390/ijerph7051953

**Published:** 2010-04-28

**Authors:** Jorge L. Ble-Castillo, María A. Aparicio-Trápala, Mateo U. Francisco-Luria, Rubén Córdova-Uscanga, Arturo Rodríguez-Hernández, José D. Méndez, Juan C. Díaz-Zagoya

**Affiliations:** 1 Centro de Investigación, DACS, Universidad Juárez Autónoma de Tabasco (UJAT), Villahermosa, Tabasco 86150, Mexico; E-Mails: rodheart@hotmail.com (A.R.H.); zagoya@servidor.unam.mx (J.C.D.Z.); 2 División Académica de Ciencias Agropecuarias, UJAT, Villahermosa, Tabasco 86280, Mexico; E-Mail: sabina52@hotmail.com; 3 Hospital General de Zona 46, Instituto Mexicano del Seguro Social (IMSS), Villahermosa, Tabasco 86060, Mexico; E-Mails: mluria@hotmail.es (M.U.F.L.); ruben.cordova@imss.gob.mx (R.C.U.); 4 Unidad de Investigación Médica en Enfermedades Metabólicas, Hospital de Especialidades, CMN Siglo XXI, IMSS, México D.F, 06703, Mexico; E-Mail: mendezf@servidor.unam.mx; 5 Unidad de Medicina Familiar N° 10, Instituto Mexicano del Seguro Social (IMSS), Xalapa, Veracruz 91000, Mexico

**Keywords:** resistant starch, banana, diabetes, obesity, insulin resistance

## Abstract

Few fiber supplements have been studied for physiological effectiveness. The effects of native banana starch (NBS) and soy milk (control) on body weight and insulin sensitivity in obese type 2 diabetics were compared using a blind within-subject crossover design. Subjects undertook two phases of 4-week supplementation either with NBS or soy milk. Patients on NBS lost more body weight than when they were on control treatment. Plasma insulin and HOMA-I were reduced after NBS consumption, compared with baseline levels, but not significantly when compared to the control treatment. Results support the use of NBS as part of dietary fiber supplementation.

## 1. Introduction

The number of people with diabetes worldwide is projected to rise from 171 million in 2000 to 366 million by 2030 [1]. This expectancy is associated with the increasing prevalence of overweight and obesity. Obesity and type 2 diabetes frequently occur together, and statistics show that 60–90% of type 2 diabetics are or have been obese [2,3]. Insulin resistance (IR) is the key mechanism unifying obesity and type 2 diabetes. IR is characterized as the tissues inability to take up glucose in response to insulin. In the course of months or years, IR is accompanied by the increase in β-cell insulin secretion and by different complications known as the insulin resistance syndrome which is associated to dyslipidemia, hypertension, hyperglycemia and cardiovascular disease [4].

When discussing strategies for metabolic control and weight loss in diabetic patients, necessarily life style modifications should be considered [5]. From the point of view of dietary habits, it has been reported that subjects with higher whole-grain intake present lower insulin concentrations [6,7], while low dietary fiber consumption is linked to a reduction in insulin sensitivity [8]. The Institute of Medicine (IOM) has defined dietary fiber as the nondigestible carbohydrates and lignin that are intrinsic and intact in plants whereas functional fiber consists of the isolated nondigestible carbohydrates to have beneficial physiological effects in human beings [9]. Accordingly, resistant starch (RS) could be considered a functional fiber by its indigestible nature and beneficial physiological effects [10]. RS escapes digestion in the human small intestine and is fermented in the colon with production of metabolically active short chain fatty acids (SCFA), [11]. Banana resistant starch belongs to a RS_2_ class which consists of native starch granules that are highly resistant to digestion by α-amylases [12]. The native banana starch (NBS) used in this study was obtained from unripe banana (*Musa Cavendish AAA*) which is cheap and produced in high levels in Tabasco State, Mexico. Starches from different sources have diverse structures and consequently different resistance to enzymatic hydrolysis. The grade of resistance to digestibility among different starches generally correlates to their amylose content. Previous studies from our group have found in this banana variety a high amylose-content and a high resistance to digestibility. Moreover a reduced glycemic and insulinic response after NBS consumption in healthy and diabetic subjects was demonstrated [13]. In the present study, we hypothesized that NBS may reduce body weight and increase insulin sensitivity in obese type 2 diabetics.

## 2. Material and Methods

### 2.1. Patients

The experimental protocol was approved by the ethical committee from the Hospital General de Zona 46 from the Instituto Mexicano del Seguro Social (IMSS). A total of 30 patients with type 2 diabetes according to the WHO criteria, were recruited from subjects attending the Hospital. The purpose of the study was explained to the participants and they gave written informed consent. Subjects were included if they had less than 10 years of type 2 diabetes diagnosis, were obese according to the WHO criterion (Body Mass Index, BMI > 30), were between 40 to 60 years of age, had maintained stable weight during three months prior to experimentation and were under the attention of a health care provider from the IMSS. Study participation did not limit the medical management they were provided. Subjects not included in this study were BMI < 30, with complications such as nephropathy or hepatic diseases, on psychiatric treatments, receiving inmmunosupressors or body reducers, with a smoking or alcoholic history and receiving insulin treatments. Seventeen subjects were taking oral hypoglycemic agents, 11 were on biguanides alone and six were taking both sulfonylureas and biguanides. A criterion for exclusion was if they modified their medical treatment during the study. All participants were examined for anthropometric measurements including height, body weight, waist and hip circumferences. Other determinations were blood pressure and fat percentage. A 24 h food record was performed by a nutritionist using a validated survey.

### 2.2. Study Design

A cross over design with two experimental periods of 4 weeks was used to compare the effects of NBS supplementation with those of soya milk. Thirty patients met the eligibility criteria, complete baseline testing and were randomly assigned to two groups of fifteen subjects to receive either 24 g of NBS dissolved in 240 mL of water per day or 24 g/day of soy milk dissolved in the same volume of water for 4 weeks. After this period all data measures were collected, groups cross over to the alternate treatment group for an additional 4 weeks period after which final measures were taken at 8 weeks. There was no wash-out period between the interventions. All participants received both supplements, and on the two phases of the experiment they were instructed not to modify their diet and exercise habits. Two women, one of each group, withdrew during the first phase due to personal reasons. The treatment compliance was supervised every week through clinical interviews in order to check the amount of consumed supplement and determine their body weight, side effects or adverse events. Further information was obtained from the patients through telephone interviews.

### 2.3. Material and Experimental Procedures

The NBS was provided by the Centro de Investigaciones Agropecuarias of the Universidad Juarez Autonoma de Tabasco. NBS was obtained from unripe (green) banana (*Musa cavendish* AAA) with a physiological age of 15 weeks obtained from a packing plant situated on Km 43.5 of the Villahermosa-Teapa road. The NBS was isolated by a previously described procedure [14]. Briefly, after washing the bananas were peeled, cut into 5–6 cm^3^ pieces, immediately rinsed in citric acid solution and then macerated at low speed in an industrial blender for 2 min. The homogenate was consecutively sieved through screens (30, 80 and 100 US mesh) and washed with distilled water; it was then centrifuged at 10,000 rpm. Sediment was further purified by washing and centrifugation. The white starch sediment was dried in a convection oven at 40–45 ºC, passed through a 100 mesh screen and stored at room temperature in sealed glass jars. The dry basis yield of starch was 60%. The proximate analysis was as follows: 3.4% of moisture content, 1.88% protein, 0.4281% fat and 0.78% ash (AOAC recommended methods 14.003, 14.057, 14.059 and 14.006). Water activity (a_w_) of NBS was measured using the Aqualab (Decagon) equipment and gave a result of 0.59.

The determination of resistance in the banana starch flour was carried out according to the enzymatic method of Goñi *et al.* [15] as follow: protein residues in the powder were hydrolyzed with pepsin under acidic conditions (pH 1.5, 40 ºC, 1 h). Then, tris-maleate buffer was added and the mixture incubated with α-amylase (37 ºC, pH 6.9, 16 h) to hydrolyze digestible starch. The hydrolysis product was eliminated and the non-digestible starch was recovered by centrifugation. After dispersing under alkaline conditions (2M KOH) to solubilize NBS, it was incubated with amyloglucosidase under acid conditions. Glucose concentration was determined by an enzymatic-colorimetric method. Resistance starch concentration of NBS was measured as percentage on a dry weight basis. A 34% of resistance was found by this method. Twenty four grams of native banana starch contained 8.16 g of resistant starch.

Soy milk powder was purchased as the commercial product Soyapac produced from whole soy beans by Colpac (Km 3, Navojoa-Sonora road, Huatabampo, Navojoa, Sonora, Mexico) with a nutritional content of 46.2% of fat, 30.8% carbohydrates, 20.5% protein.

### 2.4. Clinical Laboratory Assays

Blood samples were obtained by trained personal at the onset of the study and then after 4 and 8 weeks. Blood serum samples were immediately frozen and conserved at −70 ºC until biochemical determinations. Sera samples were analyzed for glucose, cholesterol, LDL, HDL-cholesterol and triglycerides by using an ADVIA® 1200 Chemistry System from Siemens Healthcare Diagnostics (intra-assay coefficient of variation: glucose 3.5%, cholesterol 4.9%, LDL 5.2%, HDL-cholesterol 6.1%, triglycerides 5.2%). Insulin was determined according to the method for AxSYM of Abbott based on a microassay microparticle inmunoenzymatic assay. To prevent enzymatic insulin degradation special care was taken to avoid hemolysis during the blood sampling and manipulation.

Glycated hemoglobin (HbA_1c_) was measured by a turbidimetric inmunoinhibition method according to Beckman Coulter for a Synchron CX7 Clinical System. Insulin resistance was estimated according to the Homeostasis Model Assessment (HOMA) which was calculated by the product of the fasting concentrations of glucose (mg/dL) and insulin (μU/mL) divided by 405 [16].

### 2.5. Statistical Analysis

The characteristics of the subjects are presented as mean ± SD or median and percentiles. D’Agostino-Pearson normality test was performed to assess if the data were consistent with a Gaussian distribution. Changes of endpoints after treatments at 4 weeks were expressed as change from baseline (1st treatment) and for the 2nd treatment baseline was considered the endpoint of the 1st treatment. Student’s *t* test was used for comparing variables with normal distribution or Mann-Whitney test for data from a nongaussian distribution. With the purpose to investigate the possible carry-over effects during the second treatment period we carried out the statistical analysis on the first period as a parallel design. Statistical significance was defined as p < 0.05. Calculation was performed using GraphPAD PRIZMA software version 5.01.

## 3. Results

### 3.1. Characteristics of the Patients

The baseline characteristics of 28 patients who completed the treatments are shown on Table 1. A great percentage of the obese subjects were women (85.7%), most of them housewives, 96% of them had waist circumference values over 88 cm, with a high waist-to-hip ratios, 0.873 (0.85, 0.91; median and 25th,75th percentiles). Half of the male subjects had WHR values higher than 0.95. Poor glycemic control was observed in most patients, despite their medical management, 85% had glucose values over 126 mg/dL and 42% had HbA_1c_ percentage higher than 7.0. Cholesterol levels were higher than 200 mg/dL in 60.7% of the participants and 76% had triglycerides over 160 mg/dL. In addition, 94% of patients had a sedentary lifestyle and most of them lived in rural areas and had both a low academic and socioeconomic status.

### 3.2. Dietary Treatment and Compliance

Compliance with the diet and treatment was excellent (91% for NBS and 93% for control group). No significant differences in compliance were observed between the supplemented groups. The NBS preparation was in general well tolerated, although three patients from each group reported flatulence, increased number of evacuations and soft feces during the first week of the experimental period. No other side effects or adverse events were reported by any of the participants.

### 3.3. Body Weight

Body weight and BMI were significantly reduced after NBS supplementation compared to the control group (p = 0.0021 and p < 0.0001, respectively). The NBS groups lost 1.568 kg after 4 weeks of treatment and the control group lost only 300 g (Table 2). When a statistical analysis of the first period was carried out, results were similar, body weight was reduced after NBS in comparison with baseline levels and when compared to the control treatment (p = 0.0004 and p = 0.0135, respectively).

### 3.4. Glycemic Control and Insulin Resistance

No significant changes were observed in glycemia and HbA_1c_ levels across either treatments or between treatments. However, fasting insulin concentration was significantly reduced in NBS group in comparison with baseline values (p = 0.009), but not significant when compared to the control treatment (Table 2). A small and non-significant reduction on insulin levels was observed after control treatment (p = 0.087). As it was expected, insulin sensitivity measured by HOMA was significantly increased after NBS treatment *versus* baseline values. However, it was not significant when compared to the control treatment (Table 2).

The intragroup and intergroup differences for glycemia, insulin and HOMA-I variables were similar when a statistical analysis of the first period was carried out.

### 3.5. Lipid Metabolism and Other Parameters

There were no significant effects of NBS intake on fasting triglycerides, cholesterol levels, HDL-cholesterol, LDL-cholesterol and fat body percentage. However, unexpectedly control treatment (soy milk) reduced serum triglycerides *versus* baseline values (p < 0.05, Mann-Whitney) and this effect was higher than the one observed in the NBS group (Mann-Whitney, p = 0.0121). No changes across either treatment or between treatments were observed on calcium, phosphate and hematological markers such as white blood cells, platelets and other indexes (not included in Table 2).

## 4. Discussion

In this study we have shown that NBS (24 g per day during 4 weeks) significantly lowers body weight and increases insulin sensitivity. Although there are reports on the beneficial effect of RS on the glycemic and insulin responses, there are no studies considering the effects of banana starch on diabetic obese subjects. Different studies have confirmed the beneficial effects of RS as prebiotic, hypocholesterolemiant, having hypoglycemic effects, inhibiting fat accumulation, and reducing gall stones [10].

This study was performed administering by NBS 24 g/day during 4 weeks mimicking the amount used by other groups and considering results from previous estimations obtained through food records which was 12 g/d of fiber consumption in subjects of this region. According to the current data, diets providing 30 to 50 g fiber per day from whole food sources produce lower serum glucose levels compared to a low-dietary fiber. Moreover, Dietary Reference Intakes recommend consumption of 25 g dietary fiber for adult women and 38 g for adult men, based on epidemiologic studies [9].

Although the body weight modification (1.56 Kg) could be considered small from the clinical point of view, it must be highlighted that participants did not change their diet and exercise habits during the experimentation period. In a review of many studies on the effect of dietary fiber on hunger and body composition in healthy individuals, it is reported that the average effect of 14 g/fiber supplementation per day results in a 10% decrease in energy intake and a weight loss of 1.9 Kg through about 3.8 months of intervention. Moreover, obese individuals exhibited greater reduction on body weight in comparison with lean people [17]. Comparison between studies however should be considered with caution due to differences between subjects’ health status and fiber source.

The mechanism by which fiber sources lower body weight has been focused on its effects to decrease food intake and promote satiation and/or satiety. Satiation is the satisfaction of appetite during feeding that marks the end of eating and satiety is the inhibition of hunger as a result of having eaten. Some studies support that increased fiber intake decreases hunger, provides a feeling of fullness and plays a role in the control of energy balance [18]. On the other hand, body weight reduction is associated with an increase in insulin sensitivity. This association is explained by the reduced demand on insulin production and secretion from the β-cells after losing weight. In this study, however, the small reduction on body weight after NBS might not be enough to modify the insulin demand. A better explanation is the reduced glycemic and insulinic responses induced by the NBS administration, as we have found in previous experiments after a NBS-single oral administration to healthy and diabetic subjects [13]. The chronic administration of NBS during 4 weeks might produce long-term effects which could reduce fasting insulin levels and insulin resistance on these subjects. Some authors have reported lower postprandial glucose and insulin response after resistant starch (high-amylose corn starch) supplementation to normal and overweight subjects [19]. Other reports established a reduction on insulin resistance measured with the hyperglycemic-euglycemic clamp when using type II RS (Hi-Maize 260) 30 g/day during 4 weeks [20]. In contrast, others have not found body weight reduction or fasting insulin changes after resistant corn starch 24 g/day for 21 days in obese subjects [21].

This study was not designed to investigate the biochemical mechanism of action. However, other groups have informed about the short chain fatty acids (SCFAs) and ghrelin participation in this process. SCFAs were found increased in peripheral blood and their uptake index was augmented into skeletal muscle and adipose tissue after resistant starch administration [21]. These substances have also been shown to inhibit adipose tissue lipolysis *in vivo* [22]. In addition, SCFAs augmentation has been shown to potentially increment the expression of functional proteins within the intestine because of its ability to mediate the release of glucagon-like-peptide 2 [23,24]. Another possible mechanism is the increased ghrelin hormone peripheral concentration after resistant starch. Ghrelin elevation in plasma has been linked to increased insulin sensitivity in numerous studies [25]. In addition, ghrelin inhibits lipolysis and stimulates both lipogenesis and the expression of peroxisome proliferator activated receptor γ *in vitro* (PPARγ) [26].

Limitations of the study design are its relative short duration and the lack of a wash-out period between treatments. It is evident that a residual effect of the first phase could have biased the estimate of treatment effect. However, after the statistical analysis on the first period as a parallel design, the results from the cross-over analysis were corroborated.

The lack of intergroup difference on insulin and HOMA-I could be partially explained by a soy milk small (not significant) lowering effect on both body weight and fast insulin concentration. Indeed, several studies have found beneficial effects of soy milk consumption on the glycemia homeostasis. Some authors reported that soy protein consumption lowered plasma insulin levels when compared to casein in rats [27]. Others have shown that soy phytoestrogens reduce insulin resistance improving glycemia control in postmenopausal women with type 2 diabetes [28].

No effect of NBS on lipid metabolism was observed in the present study. In contrast, soy milk supplementation reduced serum triglycerides. We do not have an exact explanation for these findings, however, the beneficial effect of soy milk supplementation on lipid metabolism is well documented in rodents and humans [29–31].

## 5. Conclusions

In this study we have demonstrated that NBS 24 g/day during 4 weeks lowers body weight and increases insulin sensitivity in a group of obese type 2 diabetics. NBS supplementation could be a cheap alternative to reduce body weight and improve glucose homeostasis on subjects with insulin resistance. Further long-term studies with larger sample size on high risk subjects are recommended.

## Figures and Tables

**Table 1.** Characteristics of the type 2 diabetic patients at baseline. Data are means ± SD or median (25th, 75th percentiles). HbA_1c_ (Glycated hemoglobin), BMI (Body Mass Index), HDL (High Density Lipoproteins).^a^ Calculated by electrical impedance (Tanita BF-350).

**Characteristics**	**Value**
Men/Women (*n = 28*)	4/24
Age (y)	51.7 ± 5.6
Body weight (Kg)	79.00 ± 16.63
Height (m)	1.50 ± 0.10
BMI (Kg /m^2^)	34.89 ± 2.32
Fasting glycemia (mg/dL)	145.94 ± 104.17
Fasting insulin (μU/mL)	14.1 (8.6, 20.30)
Waist circumference (cm)	102.5 ± 9.61
Waist to hip ratio (WHR)	0.87 (0.85, 0.91)
Body fat (%)^a^	40.93 ± 5.11
HbA_1c_ (%)	6.4 (4.5, 9.6)
Total cholesterol (mg/dL)	205.5 (187.8, 251.5)
HDL-cholesterol (mg/dL)	42.07 ± 8.60
Triglycerides (mg/dL)	227 (165.3, 311.5)

**Table 2.** Comparison of responses after consuming NBS or soy milk. Changes of endpoints after treatments at 4 weeks were expressed as change from baseline (1st treatment) and for the 2nd treatment the endpoint of the 1st treatment was used. Data are median (25th, 75th percentiles); Mann-Whitney test was used for intergroup comparisons.

	
	**NBS**	**CT**	**P value**

Body weight (Kg)	−1.2 (−1.95, −0.65)**	0.1 (−1.2, 0.7)	0.002
BMI (Kg/m^2^)	−0.59 (−0.85, −0.29)***	0.09 (−0.14, 0.37)	< 0.0001
Fasting glycemia (mg/dL)	−2.0 (−43, 39.50)	1.0 (−15.75, 19.25)	0.796
Fasting insulin (μU/mL)	−2.9 (−6.5, −0.8)**	−1.1 (−4.9,0.0)	0.420
HOMA−IR	2.21 (0.59, 3.36)*	1.12 (0.30, 2.93)	0.436
Waist−to−hip−ratio	−0.00 (−4.9, 1.36)	1.13 (−2.48, 5.92)	0.048
Body fat (%)	0.0 (−1.0, 0.0)	0.0 (0.0, 0.0)	0.442
HbA_1c_ (%)	−0.2 (−0.7, 0.15)	−0.1 (−0.6, 0.4)	0.840
Total cholesterol (mg/dL)	0.5 (−18.75, 13.75)	2.0 (−17.5, 11.50)	0.781
HDL−cholesterol (mg/dL)	0.0 (−3.25,2.75)	2.0 (−5.0, 6.0)	0.3395
Triglycerides (mg/dL)	25.0 (−36.5, 58)	−40.0 (−76.0, 16.0)*	0.012
Calcium (mg/dL)	−0.10 (−0.20, 0.20)	0.0 (−0.42, 0.30)	0.755
Phosphates (mg/dL)	0.10 (−0.25, 0.20)	−0.10 (−0.42, 0.05)	0.111

Statistical differences within group:

* p < 0.05,

** p < 0.01,

*** p < 0.0001. NBS = Native Banana Starch, CT = Control group (Soy milk).
